# Impact of the Burden of COVID-19 in Italy: Results of Disability-Adjusted Life Years (DALYs) and Productivity Loss

**DOI:** 10.3390/ijerph17124233

**Published:** 2020-06-13

**Authors:** Mario Cesare Nurchis, Domenico Pascucci, Martina Sapienza, Leonardo Villani, Floriana D’Ambrosio, Francesco Castrini, Maria Lucia Specchia, Patrizia Laurenti, Gianfranco Damiani

**Affiliations:** 1 Fondazione Policlinico Universitario A. Gemelli IRCCS, 00168 Rome, Italy; nurchismario@gmail.com (M.C.N.); marialucia.specchia@unicatt.it (M.L.S.); patrizia.laurenti@unicatt.it (P.L.); gianfranco.damiani@unicatt.it (G.D.); 2 Università Cattolica del Sacro Cuore, 00168 Rome, Italy; domenico.pascucci@outlook.it (D.P.); leonardovillani92@gmail.com (L.V.); florianadambrosio@libero.it (F.D.); fr.castrini@gmail.com (F.C.)

**Keywords:** DALY, YLL, YLD, productivity loss, COVID-19, coronavirus, pandemic

## Abstract

The WHO declared the novel coronavirus disease a pandemic, with severe consequences for health and global economic activity and Italy is one of the hardest hit countries. This study aims to assess the socio-economic burden of COVID-19 pandemic in Italy through the estimation of Disability-Adjusted Life Years (DALYs) and productivity loss. The observational study was based on data from official governmental sources collected since the inception of epidemic until 28 April 2020. DALYs for a disease combines the years of life lost due to premature mortality in the population and the years lost due to disability of the disease. In addition to DALYs, temporary productivity loss due to absenteeism from work and permanent productivity loss due to premature mortality were estimated using the Human Capital Approach. The total DALYs amount to 2.01 per 1000 persons. The total permanent productivity loss was around EUR 300 million while the temporary productivity loss was around EUR 100 million. This evaluation does not consider other economic aspects related to lockdown, quarantine of contacts, healthcare direct costs etc. The burden of disease methodology is functional metric for steering choices of health policy and allowing the government to be accountable for the utilization of resources.

## 1. Introduction

Coronaviruses are a large family of viruses known to cause diseases ranging from the common cold to more serious diseases such as the Middle East Respiratory Syndrome (MERS) and the Severe Acute Respiratory Syndrome (SARS). 

A novel coronavirus (nCoV) is a new strain of coronavirus never previously identified among humankind. In particular, the virus has been named “Severe Acute Respiratory Syndrome Coronavirus 2” (SARS-CoV-2, formerly 2019-nCoV) and it was reported, for the first time, in Wuhan, China, at the end of December 2019 [1].

The virus spreads by respiratory droplets (coughs, sneezes or talks). These droplets can be inhaled or land in the mouth or nose of a person nearby. It can also spread if a person touches a surface with the virus on it and then touches his or her mouth, nose or eyes. Most people infected with the COVID-19 virus will experience mild to moderate respiratory illness and recover without requiring special treatment (i.e., intensive care unit).

People who are older or who have existing chronic medical conditions, such as heart disease, lung disease, diabetes, severe obesity, chronic kidney or liver disease, or who have compromised immune systems may be at higher risk of serious illness. Some people may experience aggravated symptoms, such as worsened shortness of breath and pneumonia, about a week after symptoms [2].

Since 31 December 2019, when China alerted the World Health Organization (WHO) to several cases of unusual pneumonia in Wuhan, SARS-CoV-2 rapidly spread to the whole world due to the high rate of contagiousness. On 11th March 2020, the WHO declared the pandemic status [3]. 

As of 3 May 2020, more than 3.44 million cases have been reported across 187 countries and territories, resulting in more than 243,000 deaths. More than 1.09 million people have recovered [4]. 

The infectious disease was first confirmed to have spread to Italy on 30 January 2020, when two Chinese tourists in Rome tested positive for the virus and were hospitalized in isolation from 29 January. The first confirmed case of secondary transmission occurred in Codogno, in the province of Lodi, Lombardy, on 21 February 2020 [5]. 

A cluster of cases was later detected. By the beginning of March, the virus had spread to all regions of Italy. On 8 March 2020, Italian Prime Minister established the lockdown to all of Lombardy and to 14 other northern provinces. After two days, the same measures were extended to all the country, placing more than 60 million people in quarantine [6].

To better understand the enormous influence of this pandemic on Public Health, it is worthwhile to quantify the burden of the disease. There could be several ways to express the burden of an illness. Although prevalence and incidence explicate the magnitude and gravity of the problem for a given health state, there is the need to adopt summary measures which examine health outcomes and economic estimates to compare across diseases. 

The Disability-Adjusted Life Year (DALY) is one such summary measure of population health which is being increasingly used in expressing the burden due to diseases. It is a concept developed in the 1990s by the Harvard School of Public Health, the World Bank and the WHO and it is estimated by DALYs (Disability-adjusted life years). DALYs are a measure of death at different ages and of disability. One DALY can be explained as one lost year of “healthy” life and the burden of disease can be thought of as a measurement of the difference between effective health status and an ideal situation free of disease and disability old age status.

DALYs for a disease or injury derives from the sum of the Years of Life Lost (YLL) due to premature mortality in the population and the Years Lost due to Disability (YLD) for incident cases of the disease or injury [7]. 

As an additional measure of burden of disease, lost productivity could be considered as a suitable measure of economic burden of illness [8].

Productivity loss may arise from short- and long-term absences due to disability and premature death. This has a cost not only for the individual and their family, but also for society. This societal cost is lost productivity [9].

Such an analysis gives an economic evaluation which does not take into account other economic aspects related to COVID-19 (e.g., lockdown, quarantine of contacts, decrease in consumption, healthcare direct costs etc.).

The aim of the study is to assess the socio-economic burden due to the ongoing pandemic in Italy through the estimation of DALY and productivity loss. 

## 2. Materials and Methods 

In this observational study, the methodology developed by Murray and Lopez was adapted to the Italian context, aiming to quantify the relevant health impact of COVID-19 for the country.

In light of this, DALYs, calculated in an age- and gender-stratified manner, for this acute disease were estimated [10].

DALY, a summary measure of population health, combines time lost due to premature mortality (YLL) and time lived with disability (YLD).

In addition to DALYs, two types of productivity losses were estimated using the Human Capital Approach (HCA): a temporary productivity loss (TPL) due to absenteeism from work and a permanent productivity loss (PPL) due to premature mortality.

Productivity loss is a significant measure of the burden of disease, highlighting the importance of considering not only the clinical-epidemiological aspects but also the economic and social burden of that disease.

### 2.1. Study Data

The consolidated databases available from the Istituto Nazionale di Statistica (ISTAT) web site, the comprehensive data on SARS-CoV-2 in Italy since 21 February 2020 from the Istituto Superiore di Sanità (ISS) (published weekly) and data from the Istituto Nazionale della Previdenza Sociale (INPS) were used for estimating the burden of disease and the productivity losses, respectively.

### 2.2. Models Parameters 

For YLL calculation, population, number of deaths and the standard life expectancy (LE) for each age band were abstracted from the selected sources. Mid-range values for the average age at death were applied [11].

For YLD calculation, population, incident rates, duration of disease and disability weights for each age band were retrieved from the selected sources. Mid-range values for the age at onset of symptoms were applied.

Disability weight is a key component in YLD estimation. The Global Burden of Disease (GBD) study has derived disability weights for 107 health states which are the outcomes of different diseases [12].

Nevertheless, no disability weight is available for a number of health states including COVID-19. Hence, the disability weight (0.133), available for lower respiratory tract infection, the health outcome of which is comparable with the case definition of COVID-19, was adopted [13]. 

For both the temporary and permanent productivity loss, working people aged 20 to 69 years old were considered since, for the lower bound, in the previous class there were no deaths while, for the upper bound, the median between the minimum and maximum ages for the old age pension was used. 

For the TPL, number of cases, number of weeks off, hourly median wage stratified by working age class and weekly hours were retrieved from the selected sources [14]. 

The number of cases were adjusted for the number of deaths and for the number of individuals not in the working age. The number of weeks off was two according to the WHO guidelines [15]. 

The number of weekly hours was set to 40 while the hourly median wage stratified by age band was equal to EUR 10.03 for 20–29; EUR 11.39 for 30–39 and 40–49; EUR 12.03 for 50–59 and 60–69 as established by the ISTAT [16].

It was not necessary to use discounting since the time off was lower than 12 months. 

For the PPL, number of deaths, age at death, labor force participation rate, unemployment rate, retirement age, hourly median wage for each age band, weekly hours and national GDP were retrieved from the selected sources [17]. Mid-range value for the age at death was applied.

Labor force participation rate and unemployment rate was 44.9% and 10.0% respectively, as reported by the ISTAT. The retirement age was set to 69, computed as the median between the minimum and maximum retirement ages for the old age pension [18].

Productive years of life lost (PYLL_j_) for each individual belonging to a working age class W_j_ were computed as the difference between the retirement age and the average age at death for the specific working age class W_j_. The following working age classes were considered: W_1_ denoted individuals between 20 and 29 years, W_2_ between 30 and 39, W_3_ between 40 and 49, W_4_ between 50 and 59, W_5_ between 60 and 69, where j = 1…5.

Estimations of PYLL_j_ for each working age class W_j_ were as follows: PYLL_1_ were 44.5 for individuals in W_1_, PYLL_2_ were 34.5 for individuals in W_2_, PYLL_3_ were 24.5 for individuals in W_3_, PYLL_4_ were 14.5 for individuals in W_4_, and PYLL_5_ were 4.5 for individuals in W_5_, where j = 1…5.

Individual annual wage was adjusted by the labor force participation rate and unemployment rate. For each individual in working age class j, the adjusted annual wage j (AAW_j_) was defined. In particular, AAW_1_ is equal to EUR 7782 for individuals in W_1_; AAW_2_ is equal to EUR 8914.77 for individuals in W_2_; AAW_3_ is equal to EUR 8914.77 for individuals in W_3_; AAW_4_ is equal to EUR 9333.74 for individuals in W_4_; and AAW_5_ is equal to EUR 9333.74 for individuals in W_5_.

A 3% discount rate was used to discount the individual future annual wages.

### 2.3. Method of Estimating YLL and YLD

Estimates of the YLL associated to the acute respiratory infection were calculated using the YLL formula [19]:

(1) $$YLL=\frac{KCe^{ra}}{{\left(r+\beta \right)}^{2}}\left[e^{-\left(r+\beta \right)\left(L+a\right)}\left[-\left(r+\beta \right)\left(L+a\right)-1\right]-e^{-\left(r+\beta \right)a}\left[-\left(r+\beta \right)a-1\right]\right]+\frac{1-K}{r}\left(1-e^{-rL}\right)$$

where *a* is the age of death; *r* is the social discount rate; *β* is the age weighting constant; *K* is the age weighting modulation constant; *C* is the adjustment constant for age-weights; and *L* is the standard life expectancy at age of death [20].

Estimates of the years lived with disability associated to the acute respiratory infection were calculated using the YLD formula [19]:

(2) $$YLD=DW\left\{\frac{KCe^{ra}}{{\left(r+\beta \right)}^{2}}\left[e^{-\left(r+\beta \right)\left(L+a\right)}\left[-\left(r+\beta \right)\left(L+a\right)-1\right]-e^{-\left(r+\beta \right)a}\left[-\left(r+\beta \right)a-1\right]\right]+\frac{1-K}{r}\left(1-e^{-rL}\right)\right\},$$

where *a* is the age of death; *r* is the social discount rate; *β* is the age weighting constant; *K* is the age weighting modulation constant; *C* is the adjustment constant for age-weights; L is the duration of disability; and DW is the disability weight [20].

The same formulas are adopted in the Global Burden of Disease (GBD) template provided by the WHO. These formulas are based on specific parameters defined in the WHO template, where *r*, the discount rate, is 0.03, K-values are 0 when no age weights are used and 1 when age weights are used, standard age weights use a beta of 0.04 and a constant of 0.1658. K-value was set to 1 since age weights were used in the present study.

### 2.4. Method of Estimating DALY

Disability-adjusted life years were computed as the sum of the YLLs and YLDs. Absolute DALY measures were standardized to a common metric (DALY per 1000 persons) by dividing the total number of DALYs by the population and multiplying by 1000. 

Results for the following metrics were reported: total YLL; total YLL for each age-group and gender; total YLD; total YLD for each age-group and gender; total DALY; total DALY per 1000; and total DALY for each age-group and gender.

### 2.5. Productivity Losses 

For individuals, in the working age classes, suffering from COVID-19, the productivity losses were estimated adopting the HCA [21,22].

The HCA framework was implemented following the methodology proposed by Pearce et al. [14,17]. 

Estimates of individual TPL were computed as the weekly median wage for each age class by the weeks off (i.e., length of time absent from work) [14].

(3) Individual TPL=weekly median wage×weeks off,

Then, the individual temporary productivity loss was multiplied by the adjusted number of cases to obtain the total cost of COVID-19-related temporary lost productivity.

(4) total TPL=individual TPL×adjusted number of COVID−19 cases,

Estimates of individual PPL for a specific working age class $W_{j}$ were calculated as the sum of the discounted individual annual wages, for each PYLL_j_, adjusted by labor force participation rate and unemployment rate.

(5) $$Individual\;PPL=\sum_{t=0}^{PYLL_{j}}\frac{AAW_{j}}{{\left(1+r\right)}^{t}},$$

Annual wage was adjusted according to the following formula [17]:

(6) (annual wage×labor force participation rate×(1−unemployment rate)),

Then, the individual productivity loss was multiplied by the number of deaths to obtain the total cost of COVID-19 related permanent lost productivity.

(7) total PPL=individual PPL×number of deaths,

Results for the following metrics were reported: individual cost of temporary lost productivity; total cost of temporary lost productivity; cost of permanent lost productivity per death; total cost of permanent lost productivity; and total cost of permanent lost productivity as a share of national GDP.

## 3. Results

COVID-19 affected 107 provinces in 20 regions in Italy during the first quarter of 2020.

On 28 April 2020, the total population at risk of infection was 60,359,546 and the number of SARS-CoV-2 cases were 199,470 while the number of deaths was 25,215 as reported by the ISS [23]. The overall incidence per thousand population was estimated to be 3.30 and 80% of incident cases were concentrated in the North of Italy. 

The crude mortality rate (CMR) per thousand population was 0.42 while the overall case fatality rate (CFR) of persons with confirmed COVID-19 in the Italian population was 12.76% [23].

### 3.1. DALYs

On 28 April 2020 the estimates of the burden of disease due to COVID-19 in Italy were 121,449 DALYs as a discount rate of 3%, divided in 82,020 for males and 39,429 for females. Stratifying this composite indicator, total YLL totaled 81,718 in males and 39,096 in females while total YLD equaled 302 in males and 333 in females (Table 1). 

**Figure 1:**
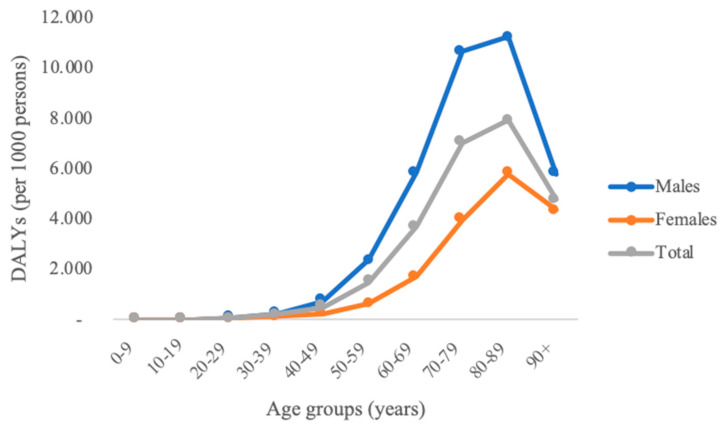
Age-specific DALYs rate per 1000 persons among males and females in Italy.

The DALY rate in Italy was 2.01 DALYs per 1000 persons, with the estimated burden of disease being the highest among people aged 80–89 years (Figure 1).

### 3.2. Productivity Losses 

In Italy in 2020, the total cost of lost productivity due to absenteeism from work was around EUR 100 million for all the working age classes (Table 2).

The total cost of lost productivity due to COVID-19 premature mortality for all the working age classes was around EUR 300 million and its impact on the national GDP was estimated to be 0.17% (Table 3).

**Figure 2:**
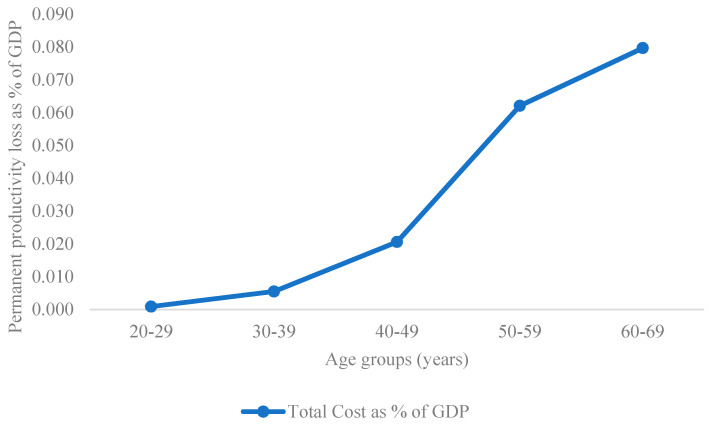
Permanent Productivity Loss as a % of GDP for each working age class.

As for DALYs, the major impact on GDP derives from the oldest working age class (Figure 2).

## 4. Discussion

Burden of disease, based on the DALY metric, has been widely used to estimate the impact of lower respiratory infections since its development in the 1990s [24,25].

The current study found that the high burden from COVID-19 was mainly due to mortality. Almost 99.48% of DALYs was due to YLL. 

Gender distribution showed that YLL were higher in males than those in females, except for the last age band (i.e., 90+). YLD were higher in males than those in females only in the first, seventh and eight age band (i.e., 0–9; 60–69; 70–79). 

Although sex-disaggregated data for COVID-19 show equal numbers of cases between men and women so far, there seem to be sex differences in mortality and vulnerability to the disease. Emerging evidence suggests that more men than women are dying, potentially due to sex–based immunological or gendered differences, such as patterns and prevalence of smoking. However, current sex-disaggregated data are incomplete, cautioning against early assumptions [26].

Evidence from Sharma et al. suggests that a report on COVID-19-related deaths from Italy showed higher deaths for men than women across all age groups [27].

An age-wise distribution highlighted that people aged 70–79 years contributed to the highest number of DALYs accounting for 34.55% of the total DALYs. However, the highest DALY rate was detected in people aged 80–89 years. 

In accordance with the present results, population-based studies have demonstrated that the prevalence of comorbidities and number of comorbid conditions increase with age [28].

A strong relationship between COVID-19 and existing comorbidities has been reported in the literature [29]. Indeed, as mentioned in the report about the characteristics of SARS-CoV-2 patients dying in Italy by the ISS, hypertension, type II diabetes, and ischemic heart disease are the most frequent pre-existing pathologies among patients with a frequency of 69.2%, 31.8%, and 28.2%, respectively. Additionally, the proportion of individuals with one or two pathologies is 14.5% and 21.4%, respectively, while those with three or more conditions account for 60.3%, highlighting the high number of comorbidities in elderly people [30].

Along with the presence of multiple conditions, these findings are also supported by the fact that a high proportion of the individuals admitted to the intensive care unit (ICU) is elderly [31].

The highest number of DALYs among elderly people may be further confirmed by evidence in the literature showing that an increasingly number of older people are dying in care homes and suggesting that unprepared home cares are new COVID-19 hotspots, struggling to secure safe lockdown for elderly residents at risk of severe and fatal SARS-CoV-2 infections [32,33].

The economic perspective on the COVID-19 burden, given by the estimation of the productivity losses, complements existing population health metrics underlying the importance of considering its impact on the productive capacity of the labor force.

The adopted methodology, based on the Human Capital Approach, is a validated framework to calculate lost productivity for both acute and chronic conditions. 

Prior studies have noted the importance of estimating the economic burden of lower respiratory tract infections (LRTI) [34,35].

The findings of the current study show that productivity loss was largely due to premature mortality. Indeed, the number of deaths is ten times higher in 60–69 working age class than in the 40–49 one. As a consequence, the oldest age class has the highest impact although the number of productive years of life lost is lower than that of the younger age classes. 

This is translated into a total lost productivity of almost EUR 143 million for the 60–69 age group which represents 0.08% of the national GDP.

Even though the lost productivity due to absenteeism is lower than the one due to premature mortality, its impact is significant both at the individual and societal level. In other words, suffering from COVID-19 leads to an average individual loss among working age classes of approximately EUR 915 and a societal loss of roughly EUR 100 million.

### 4.1. Main Implication

The rapidly changing epidemiology presents a challenge to which the National Health Service must respond. There is a need to focus on preventing as well as treating the conditions causing the greatest disease burden and the greatest demand for our health and care services [36]. The healthcare metric employed in this study can be adopted to steer choices of decision makers in the establishment of priorities for research and policy in case of emergency conditions [37]. In this light, information on the economic impact of illness and health problems is intended not to replace but rather to supplement the epidemiological information on health problems of populations [38].

### 4.2. Strengths and Limitations

Data collected in this study refer to the first phase of lockdown imposed by the Italian government following the rapid spread of the virus which began at the end of February, in the North of Italy, and ended up on 4 May 2020, corresponding to the beginning of the second phase, with a slackening of precautionary measures previously adopted on 8 March 2020.

The analysis is novel in that it estimates the burden of COVID-19 in Italy. The study is based on epidemiological and socio-economic data retrieved from official and governmental sources such as the ISS [39], ISTAT [40], INPS [41], and the Italian Ministry of Health [42]; nonetheless, epidemiological data are grounded on the guideline that swabs are done only to symptomatic or suspect individuals [43,44,45]. Furthermore, there is an additional amount of 11,600 deaths for which the cause of death is not specified [46].

Moreover, DALYs estimates refer only to the acute phase of the disease inasmuch as the pandemic is still ongoing and the several sequelae and chronicities, to which COVID-19 could be correlated, are not well defined so far. Indeed, the probability to develop sequelae or chronic conditions is high and it is already described in the scientific literature [47,48,49]. 

In the estimation of YLDs, the disability weight was assumed as an acute lower respiratory infection since a specific value for COVID-19 is still not available, as performed by Gaunt et al. [11] for Influenza and other coronaviruses. Nevertheless, the adopted formulas are endorsed by the WHO.

Our evaluation, deliberately focused on the productivity loss, gives a quantitative characterization of the burden of disease without, however, considering other aspects related to COVID-19 (e.g., lockdown, quarantine of contacts, decrease in consumption, healthcare direct costs etc.).

In the calculation of temporary productivity loss, the length of time absent from work varies highly from one person to another and from the speed in doing swabs to confirm the clinical recovery [50]. 

For both the temporary and permanent productivity loss, the labor force participation rate and the unemployment rate are not age-dependent as it was not possible to match data due to a different age stratification. Regarding the hourly wage, differently from what is stated above, ISTAT reports different age groups from those reported by ISS; however, we solved the issue by adapting their age bands (i.e., 15–29; 30–49; and 50 and over) to ours (i.e., 20–29; 30–39; 40–49; 50–59; and 60–69). 

The retirement age, used for the calculation of the permanent productivity loss, is assumed as a median since the Italian pension system, based on several working characteristics for each individual, allows different retirement ages.

Notwithstanding, the adopted formulas are referenced in the scientific literature.

Further studies, adopting the same metrics, are needed to compare the Italian scenario with the international ones.

## 5. Conclusions

The new coronavirus emergency is threatening societies at their core, affecting the lives and livelihoods of millions of people worldwide with devastating impacts on social and economic aspects. 

Indeed, in Italy in the first quarter of 2020 the excess deaths are 25,354, of which 54% are COVID-19 diagnosed deaths (13,710) [46].

The characterization of the Burden of Disease with DALYs and the Productivity Loss metrics is essential to provide support to allow the central government to be accountable for the financing and allocation of resources aimed at the planning of health policies designed to prevent emergency events of such magnitude.

## Figures and Tables

**Table 1:** Years of lost life (YLL), years lost due to disability (YLD), and disability-adjusted life years (DALY) attributable to COVID-19 stratified by age and gender in Italy.

Age Group	Total YLL, YLD, and DALY for Males			Total YLL, YLD, and DALY for Females		
	YLL	YLD	DALY	YLL	YLD	DALY
0–9	36.28	1.78	38	36.48	1.55	38
10–19	0.00	6.13	6	0.00	6.15	6
20–29	197.39	24.86	222	66.49	32.36	99
30–39	863.04	35.18	898	466.14	42.66	509
40–49	3479.70	49.30	3529	1192.04	66.57	1259
50–59	10,749.30	63.52	10,813	3016.28	70.07	3086
60–69	20,507.93	50.13	20,558	6476.51	31.98	6508
70–79	29,000.47	40.32	29,041	12,894.20	28.48	12,923
80–89	15,671.33	25.95	15,697	12,497.19	35.21	12,532
90+ Total	1212.48 81,718	5.00 302	1217 82,020	2450.85 39,096	17.52 333	2468 39,429

**Table 2:** Estimated temporary productivity loss per age groups calculated with the Human Capital Approach (HCA) (2020 euros).

Age Group	Number of Adjusted Cases *	Individual Cost of TPL	Total Cost of TPL
20–29	10,369	EUR 802.40	EUR 8,320,085.60
30–39	14,858	EUR 919.20	EUR 13,657,473.60
40–49	25,420	EUR 919.20	EUR 23,366,064.00
50–59	35,068	EUR 962.40	EUR 33,749,443.20
60–69	25,153	EUR 962.40	EUR 24,207,247.20
Total	110,868		EUR 103,300,313.60

* The number of cases were adjusted by the number of deaths and for the number of individuals not in the working age.

**Table 3:** Estimated permanent productivity loss per age groups calculated with the HCA (2020 euros).

Age Group	Number of Deaths	Individual Cost of PPL	Total Cost of PPL	Total Cost of PPL as% of GDP
20–29	8	EUR 198,617.16	EUR 1,588,937.30	0.0009
30–39	49	EUR 200,515.55	EUR 9,825,261.95	0.0055
40–49	224	EUR 164,212.36	EUR 36,783,568.10	0.0206
50–59	918	EUR 120,848.52	EUR 110,938,945.80	0.0621
60–69	2727	EUR 52,199.42	EUR 142,347,805.00	0.0796
Total	3926		EUR 301,484,518.15	0.1686
